# Melanocortin therapies to resolve fibroblast-mediated diseases

**DOI:** 10.3389/fimmu.2022.1084394

**Published:** 2023-01-30

**Authors:** Natalya Khodeneva, Michelle A. Sugimoto, Camilla S. A. Davan-Wetton, Trinidad Montero-Melendez

**Affiliations:** The William Harvey Research Institute, Queen Mary University of London, London, United Kingdom

**Keywords:** fibroblasts, melanocortin, drug development, resolution of inflammation, fibrosis

## Abstract

Stromal cells have emerged as central drivers in multiple and diverse diseases, and consequently, as potential new cellular targets for the development of novel therapeutic strategies. In this review we revise the main roles of fibroblasts, not only as structural cells but also as players and regulators of immune responses. Important aspects like fibroblast heterogeneity, functional specialization and cellular plasticity are also discussed as well as the implications that these aspects may have in disease and in the design of novel therapeutics. An extensive revision of the actions of fibroblasts on different conditions uncovers the existence of numerous diseases in which this cell type plays a pathogenic role, either due to an exacerbation of their 'structural' side, or a dysregulation of their 'immune side'. In both cases, opportunities for the development of innovative therapeutic approaches exist. In this regard, here we revise the existing evidence pointing at the melanocortin pathway as a potential new strategy for the treatment and management of diseases mediated by aberrantly activated fibroblasts, including scleroderma or rheumatoid arthritis. This evidence derives from studies involving models of *in vitro* primary fibroblasts, *in vivo* models of disease as well as ongoing human clinical trials. Melanocortin drugs, which are pro-resolving mediators, have shown ability to reduce collagen deposition, activation of myofibroblasts, reduction of pro-inflammatory mediators and reduced scar formation. Here we also discuss existing challenges, both in approaching fibroblasts as therapeutic targets, and in the development of novel melanocortin drug candidates, that may help advance the field and deliver new medicines for the management of diseases with high medical needs.

## 1 Introduction

All tissues and organs in our bodies contain fibroblasts, a type of spindle-shaped cells which produce the connective tissue that provides structural support to the parenchymal cells (1). Here, their main role is to secrete the components of the extracellular matrix (ECM), mostly composed of collagens, fibronectin, elastin, laminins, proteoglycans and microfibrillar proteins (2). However, much has progressed since the 19^th^ century when Virchow, Ziegler and Ramon y Cajal identified and provided the first clues about the existence and the functions of these cells (1). Whilst historically fibroblasts were defined by and studied mostly because of their role in wound healing, we now know that the structural role is only one of the many functions that fibroblasts can display, as discussed later.

Although a single name is used to refer to fibroblasts, they represent a highly heterogeneous population, with different subtypes identified depending on the specific tissue, their location within each tissue, pathophysiological status and cellular origin. This has become evident mostly in recent years with the advent of single-cell RNA sequencing techniques, which have helped to unravel the vast heterogeneity and plasticity of this cell type (3–5). Remarkably, single-cell based techniques have started to reveal something more important than the specific markers that define each subpopulation, that is the functional specialization of the different subtypes and how they differentially contribute to homeostasis and disease (6–8). The translational impact of this new breadth of knowledge is also notable as opportunities for the selective targeting of specific pathogenic populations, as well as pro-resolving ones, could now be developed.

Another important conceptual shift in the field of fibroblast research is the recognition that these cells can be ‘drivers’ of disease, rather than mere ‘responders’ to the inflammatory or pathogenic microenvironment. For example, in rheumatoid arthritis (RA), cartilage and bone destruction are mediated by fibroblasts that “fail to switch-off”, leading to persistent activation of immune cells, resulting in chronic inflammation (9, 10). Thus, it is proposed that strategies targeting the fibroblasts aimed at promoting pro-resolving mechanisms, i.e., the mechanisms that actively lead to the termination of the inflammatory response (11, 12), could be used for the treatments of diseases mediated by abnormally activated fibroblasts.

One of the endogenous pro-resolving pathways that has extensively been studied at the preclinical and clinical levels, and indeed has reached approval for use in humans, is the targeting of the melanocortin pathway (13). This system comprises a family of five membrane receptors and four endogenous agonists which, among other functions, have demonstrated anti-inflammatory and pro-resolving actions in various models of disease, including arthritis (14–16), gout (17), intestinal inflammation (18, 19), atherosclerosis (20), uveitis (21), transplant rejection (22), or neuroinflammation (23) among others. Their role in controlling fibroblast activation have also been studied, and this includes *in vitro*, *in vivo*, and clinical investigations. In this review we collect the existing evidence supporting the therapeutic potential of melanocortin therapies to control fibroblast mediated diseases and we analyse the trends and directions that may help for the successful translation into the clinics of this new class of drugs.

## 2 Fibroblasts in health and disease

### 2.1 ‘Structural side’ of fibroblasts

The structural functions of fibroblasts extend beyond the passive provision of a scaffold to sustain other cells within an organ. The components of the matrix secreted by fibroblasts can directly interact with membrane receptors, like integrins and syndecans, and initiate intracellular signalling pathways which play important roles in various processes like organ morphogenesis, cell migration or mechanotransduction (24, 25). Of note, the contribution of cell-matrix interactions to homeostasis is the reason why 3D organoid models incorporating ECM or fibroblasts in their composition provide a more reliable system than monolayer cultures (26).

Fibroblasts are involved not only in the deposition of ECM components, but also in their appropriate remodelling through crosslinking and proteolysis. These actions provide the fibroblasts a key role in orchestrating the process of wound healing and repair upon tissue injury (27). Thus, when blood comes into contact with collagen in the ECM of tissues, it triggers platelet activation, initiating the clotting cascade. Other ECM components crucial at this stage are fibrin and fibronectin, which form a temporary plug to support the wound. Immune cells, initially neutrophils, are then recruited to clear dead cells and pathogens. Fibroblasts lead the next phase characterised by the accumulation and activation of these cells in the wound bed and the release of large amounts of type III collagen at first, which is later replaced with stronger type I collagen. These activated fibroblasts, typically referred as myofibroblasts due to the expression of alpha-smooth muscle actin (αSMA), direct the contraction of the deposited ECM bringing the wound edges together. During the remodelling phase, which can last from weeks to years, the collagen, originally released in a disorganised way, is realigned along tension lines, and crosslinked to achieve the desired tensile strength. Pathologies that derive from a dysregulated action of fibroblasts during the wound healing process include chronic wounds, excessive scarring or fibrotic pathologies (28).

A distinct structural role of this cell type is exemplified by synovial fibroblasts. Although they also provide structural support to the synovial tissue that lines the joint capsule, a key role of synovial fibroblasts is the provision of nutrients to the avascularised cartilage and lubrication to the joints by the release of hyaluronan and lubricin into the synovial fluid allowing the movements of the joints (29). As discussed later, synovial fibroblast dysfunction can also drive pathological states in conditions like rheumatoid arthritis.

In summary, all the actions discussed above can be grouped into the ‘structural side’ of fibroblasts. However, these cells also exert direct immune actions by themselves or *via* the interaction with immune cells, with important implications in disease. These actions will be summarised next.

### 2.2 ‘Immune side’ of fibroblasts

Besides their well characterised structural role, it is now clear that fibroblasts can directly exert actions typically attributed to immune cells. For example, dermal fibroblasts have membrane expression of Toll-like receptors (TLRs), which actively engage in the body’s defence against invading pathogens and therefore suggest an innate immune role for fibroblasts (30). Likewise, cytoplasmic nucleotide-binding oligomerisation domain containing proteins 1 and 2 (NOD1, NOD2), involved in the intracellular recognition of bacteria, have been found on dental pulp fibroblasts (31). Fibroblasts can also sense bacterial metabolites through the expression of xenobiotic receptors, of relevance in the gut where this process may modulate their inflammatory and pro-fibrotic properties (32). Furthermore, fibroblasts can also release anti-microbial peptides such as defensins and cathelicidins (33, 34), clearly suggesting that fibroblasts can elicit direct immune responses against microorganisms at least in highly colonised tissues like the skin, oral cavity, intestinal lumen and the eye. Altogether, this indicates that fibroblast functions may be highly specialised depending on tissue needs.

Fibroblasts can also produce a vast array of pro-inflammatory mediators including cytokines, chemokines and growth factors like TNF-α, IL-1β, IL-6, IL-8, CCL-1, CCL-2, CXCL-5, GM-CSF or G-CSF, leading to the recruitment and activation of immune cells and contributing to processes like angiogenesis, fibrogenesis and modulation of apoptosis resistance. Fibroblasts express histocompatibility leukocyte antigen DR (HLA-DR) molecules, the activation of which can contribute to the modulation of T cell phenotypes as well as to the release of pro-inflammatory cytokines (35). Interestingly, in addition to pro-inflammatory mediators, fibroblasts also express receptors and release molecules with anti-inflammatory and pro-resolving actions like IL-10, resolvins and melanocortin peptides (see Sections 3 and 4), providing a distinct framework for the therapeutic targeting of fibroblast-mediated diseases.

Another well-known immune role of fibroblasts is attributed to those cells found in the lymph nodes, referred to as fibroblastic reticular cells, which tightly control adaptive immune cell homeostasis, for example by producing IL-7 to support naive T cells (36). This represents another example denoting fibroblast functional specialization.

The immune role of fibroblasts is also driven by their ability to interact with immune cells and influence their responses. Interestingly, this interaction is usually bidirectional, whereby immune cells also influence fibroblast behaviour (37). For example, using *in vitro* cultures, Zhou et al. proposed the notion of a ‘stable two-cell system’ between macrophages and fibroblasts, where fibroblasts secrete colony stimulating factor 1 (CSF-1), essential for the survival of macrophages, and in turn macrophages produce platelet-derived growth factor (PDGF) that is key in promoting the proliferation and maintenance of fibroblast populations (38, 39). This fibroblast-macrophage crosstalk has also been observed in pathological contexts like fibrosis, where fibroblasts induce the recruitment and activation of macrophages through CCL-2 and CSF-1, while macrophages in turn activate fibroblasts by releasing transforming growth factor beta 1 (TGF-β1) and IL-6 (39). Similarly, fibroblasts and macrophages co-exist and interact in both the lining and sub-lining layers of the synovium. While fibroblasts provide support, nutrients and lubrication as mentioned earlier, the role of tissue-resident macrophages in the synovium during physiological conditions is not well known (40). To add further complexity to the interactions between these two cell types in the synovium, Alivernini et al demonstrated first, that these interactions and their consequences may depend on specific subsets of macrophages, and second, that these interactions can not only reinforce inflammatory status, but rather the opposite, that is to promote repair responses in fibroblasts *via* the release of pro-resolving lipids like resolvin D1 (7).

An important aspect that emerges from the understanding of the direct immune actions exerted by fibroblasts and the immune-stromal crosstalk is the appreciation that targeting the fibroblast may be as important as targeting the immune cells. This suggests new therapeutic strategies to control multiple pathological conditions that, although they display an immune component, are now known to be driven by abnormally activated fibroblasts. Examples of these conditions will be discussed next.

### 2.3 Fibroblasts are active drivers of disease

**Figure 1:**
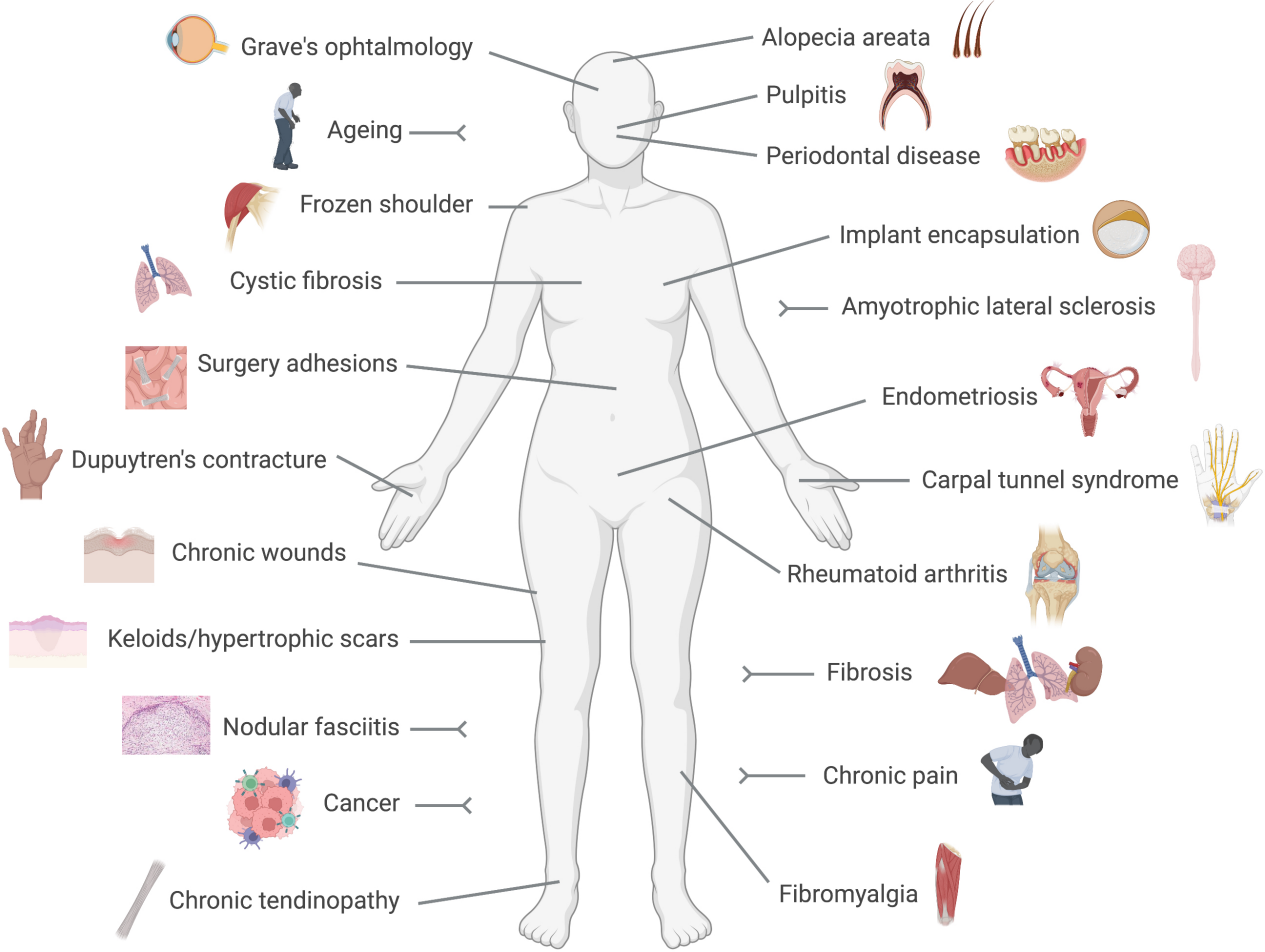
Fibroblast-mediated diseases. Conditions in which a pathogenic driving role for fibroblasts has been reported are represented in this cartoon. These include numerous diseases with a clear pro-fibrotic component, like fibrosis, Dupuytren's contracture or implant encapsulation, and conditions in which the immune side of fibroblasts results pathogenic like rheumatoid arthritis or cancer.

Fibroblasts are no longer considered relatively quiet cells, resting in tissues producing and releasing matrix components. Indeed, because of their presence in almost every tissue and organ in the body, it is not surprising that fibroblasts are now implicated in multiple and diverse conditions ranging from well-known diseases like cancer, rheumatoid arthritis or fibrosis, to poorly understood ones like fibromyalgia, chronic pain or thyroid eye disease, as we will discuss below (**Figure 1**). The recent acceptance of the fibroblast as an active driver of disease, and consequently as a therapeutic target, may bring innovative strategies to treat and manage those conditions.

In rheumatoid arthritis, the pathogenic role of fibroblasts is very well established. While initially activated by their pro-inflammatory microenvironment, at a certain stage, fibroblasts acquire an epigenetically imprinted aggressive phenotype switching from responders to drivers, and hence switching the disease from acute to chronic (41). Their pathogenic actions involve the secretion of pro-inflammatory cytokines, chemoattractants and pro-angiogenic factors, the release of metalloproteases involved in the degradation of cartilage and bone and the display of apoptosis-resistant and invasive behaviour (41, 42). The active role of synovial fibroblasts was also demonstrated *in vivo*. Mice that lack an organised synovial lining layer due to the deficiency of cadherin-11 are indeed resistant to the development of arthritis, demonstrating a causal role of fibroblasts in the disease (43). Different synovial fibroblast subpopulations with functional specialization have been identified within the synovium. Croft et al recently showed that while FAP^+^THY1^+^ cells are mainly mediators of inflammation, the destruction of joint tissues is mostly mediated by FAP^+^THY1^-^ fibroblasts (8). Interestingly, studies using a mouse model of joint inflammation showed that fibroblasts can acquire the ability to transmigrate (and transfer their imprinted aggressiveness) to distant joints, suggesting not only a local role in driving tissue destruction but also in the progression and spreading of the disease to unaffected joints (44).

The driving role of fibroblasts in cancer is also well studied. Characterisation of cancer-associated fibroblasts (CAF) heterogeneity has significantly contributed to understanding the diverse phenotypes and functions that fibroblasts can assume during this disease. CAFs display a range of tumour-supporting functions, including migration, metastasis, angiogenesis, resistance to chemotherapy and immunosuppression (45, 46). However, the diverse pro-tumorigenic functions that CAFs can play do not belong to a unique cell population. For example, work from Öhlund et al in pancreatic ductal adenocarcinoma uncovered the co-existence of CAF subtypes bearing either tissue-remodelling or immunosuppressing features, termed myofibrotic CAF (myCAF) and inflammatory CAF (iCAF), respectively (47). The authors also postulate that, besides the demonstrated pro-tumorigenic role of CAFs, previous attempts at targeting fibroblasts to treat cancer may have been unsuccessful because they mainly targeted the myCAF subpopulation while leaving other subsets unaffected.

The prototype of fibroblast-mediated diseases is undoubtedly fibrosis. Fibrosis is typically referred to as a *wound that never heals*, where the involvement of fibroblasts is mostly derived from the dysregulation of their 'structural side', in contrast to the previous examples, where the 'immune side' plays a more prominent role. In fibrosis, the healing process continues unresolved to the point of causing scarring and impairment of tissue function. In general, although with exceptions, activated endothelial cells act as the initial stimulus, causing the subsequent recruitment of leukocytes which release a host of pro-fibrotic cytokines like IL-6 and TGF-β1, which act on local fibroblasts to create a fibrogenic environment, causing the transition from inactive into aberrantly activated αSMA expressing myofibroblasts (48). These myofibroblasts have a significant role in the development of fibrosis, secreting high levels of ECM proteins, and perpetuating fibroblast activation through the overexpression of TGF-β1. Moreover, the myofibroblast population exhibits a higher level of resistance to apoptosis (49), contributing to the continuous presence of ECM-producing fibrotic fibroblasts. The eventual organ dysfunction results from the progressive replacement of functional parenchymal cells in the affected organ with disorganised scar tissue. As for rheumatoid arthritis synovial fibroblasts, stable epigenetic modifications particularly on fibroblast genes like *ACTA2* and *THY1* (encoding for αSMA and CD90, respectively), also contribute to the progression of fibrosis (50). Fibrosis can manifest in many organ systems, like skin fibrosis including keloids and hypertrophic scars (51); pulmonary fibrosis, caused by exposure to pollutants like asbestos, chemotherapy agents or bacterial and viral infections (52–54); liver fibrosis, associated with alcoholism, fatty liver disease or viral infection (55); cardiac fibrosis upon myocardial infarct (56); intestinal fibrosis, associated with inflammatory bowel disease (57); dystrophic epidermolysis bullosa caused by a mutation in the collagen VII gene (58), or myelofibrosis, which may be caused by mutations in the gene encoding for Janus kinase 2, *JAK2*, and affects the bone marrow (59). In other cases, the aetiology is not known, like for example in idiopathic pulmonary fibrosis (60), affecting mainly the elderly, or in systemic sclerosis, which presents with an autoimmune component and affects the skin as well as internal organs (61, 62). Notably, life expectancy is dramatically lowered in patients affected with fibrotic diseases, while almost no treatments currently exist.

Chronic wounds are also wounds that fail to heal, but unlike fibrosis, where fibroblasts get 'stuck' in a perpetual proliferative and collagen deposition phase (i.e. remodelling), chronic wounds are characterised by the failure to progress beyond the initial phases of wound healing of haemostasis and inflammation. A deficiency rather than an excess of fibroblast activity is involved, and may be attributed to their decreased proliferative, migratory and contractile capacity (63). Interestingly, a better understanding of the signals that drive the excessive remodeling in fibrosis may help to inform on novel strategies to activate this phase in chronic wounds (64). Ageing and cellular senescence in fibroblasts might also contribute to chronic wounds, by a mechanism proposed to be telomere independent in both chronic venous leg ulcers, typically associated with age (65), and in diabetic ulcers, in which senescence is believed to be induced by sustained hyperglycemia (66). Although senescent cells appear naturally and promote wound healing in physiological conditions (67), it is believed that their accumulation in chronic wounds due to the inefficiency of the immune system and subsequent persistent release of proinflammatory mediators [e.g. IL-6, IL-8, CCL8, MIF, etc (68)] may turn these cells pathogenic in this context (69).

Linked with senescence is also the involvement of fibroblasts in ageing. Cell division causes the gradual shortening of chromosomal ends, named telomeres, triggering cellular senescence when a critical point is reached. This protective endogenous anti-cancer mechanism prevents the spreading of DNA-damaged cells (70–72). Senescent fibroblasts present with reduced collagen deposition, increased proteolytic activity and reduced proliferation. During ageing these cells accumulate in tissues, likely due to the inefficiency of the aged immune system to detect and clear them (73), and they release pro-inflammatory mediators that cause tissue damage and alter tissue homeostasis and function (74). This *un-resolved* senescence program (72), i.e., not culminating in clearance, is believed to be responsible for the damaging effects of senescent cells. However, senescence in fibroblasts can also enhance and accelerate wound healing and reduce fibrosis (67, 75–78). The different outcomes of fibroblast senescence are highly dependent on multiple contextual aspects (72) such as age, specific tissue, immune system contribution, and very likely but largely unexplored, the specific fibroblast subpopulations involved.

The fascia is a thin layer of connective tissue composed mostly of collagen, present beneath the skin and surrounding organs, vessels or muscles, in which the predominant cell type is the fibroblast. Not surprisingly, fascia fibroblasts also drive the development of various diseases. For example, nodular fasciitis is a benign tumour tissue driven by αSMA expressing myofibroblasts, caused by gene rearrangements involving the gene ubiquitin-specific protease 6, *USP6* (79, 80). A condition known as Dupuytren's contracture develops in the fascia of the palms. Fibroblast activation leads to the formation of palmar nodules which turn into fibrotic cords that extend into the digits, causing their permanent flexion and deformities with consequent impairment of hand function (81). Surgery adhesions are fibrotic scars mediated by tissue-resident fibroblasts of the fascia, which develop in 50-90% of abdominal operations, representing a major health burden (82, 83). These fibroblasts are characterised by increased expression of αSMA and vimentin, and increased collagen deposition. It was demonstrated in animal models that treatment with anti-TGF-β1 blocking antibodies prevents the formation of abdominal adhesions, highlighting the prominent driving role of fibroblasts in this condition (84). Inflammation of the fascia is also involved in the pathogenesis of fibromyalgia, a very debilitating condition causing widespread pain, tiredness and disability. It has been hypothesised that fibroblasts in the fascia surrounding muscles are the source of pro-inflammatory mediators causing inflammation of the fascia (85). It was then proposed that inflammation of the fascia is the source of the peripheral nociceptive input that leads to central sensitization. Strategies targeting hyperactivated fibroblasts may offer novel opportunities to manage fibromyalgia.

Other conditions associated with debilitating pain in which fibroblasts contribute include chronic pain, in which fibroblasts are responsible for releasing proalgesic mediators and sustaining inflammatory responses (86), or carpal tunnel syndrome, a peripheral neuropathy characterised by fibrosis occurring in the subsynovial connective tissue in the carpal tunnel, with fibroblast hyperplasia, disorganised collagen deposition and increased expression of TGF-β1 pathway components (87). Therapies using typical antifibrotic strategies like targeting this profibrotic pathway have shown promising preliminary results in this syndrome (88, 89). Fibroblasts also contribute to the pathogenesis of frozen shoulder (90), and chronic tendinopathy (91).

The role of fibroblasts in the oral cavity is also well studied. The loss rather than the lack of resident periodontal ligament fibroblasts is a feature of periodontal disease (92). On the other hand, another type of fibroblast termed pulp fibroblasts, residing in the soft inner tissue inside a tooth, is responsible for the recognition of pathogens and initiation of the inflammatory response in a condition called pulpitis (31). Another interesting example is represented by the role of orbital fibroblasts in the pathogenesis of a condition called Grave's ophthalmopathy, associated with a form of autoimmune hyperthyroidism. Excessive immune and pro-fibrotic activity of these fibroblasts may be mediated by autoantibodies against the thyroid stimulating hormone receptor (TSH-R), which are expressed in orbital fibroblasts (93). Endometriosis is also associated with a dysregulation of endometrial mesenchymal fibroblasts (94), leading to endometrial tissue growth outside the uterus that can result in infertility.

Fibrotic encapsulation of silicone implants used in aesthetic or reconstructive medicine (e.g., mammoplasty, rhinoplasty) represents another important medical need. Implantation of a foreign body causes an inflammatory response followed by the release of TGF-β1 and activation of myofibroblasts, which deposit large amounts of collagen and cause contraction of the tissues, leading to implant deformities and pain (95, 96). However, this area is recently attracting more attention with the development of wearable medical devices like continuous glucose monitoring systems (CGM). It was recently reported that strategies to reduce fibrotic encapsulation of CGM electrodes may improve their sensitivity and provide a more accurate measurement of glucose levels (97).

The list of conditions where fibroblasts actively contribute to pathogenesis seems endless. Other examples include cystic fibrosis, where high levels of TGF-β1 and myofibroblasts are present (98), alopecia areata where, on the contrary, a deficiency of dermal papillae fibroblasts' functions is observed (99), and amyotrophic lateral sclerosis characterised by abnormal perivascular fibroblast activity preceding the onset of the disease (100).

Collectively, the above review of diseases driven by fibroblasts emphasizes an existing large clinical need. Fibroblasts can drive diseases all over the body (**Figure 1**), for many of which no therapies are currently available. However, multiple strategies to target the fibroblast from different angles are being developed, as discussed next.

### 2.4 Fibroblasts as therapeutic targets

Fibroblasts do more than secreting matrix. They proliferate, they migrate and invade tissues, they contract, they induce and enhance inflammation, they communicate to other cells to share messages and they extend their own lifespan. These activities, however, offer opportunities to target the fibroblast using a variety of approaches.

One of the most studied strategies consists of preventing or reversing the fibroblast-to-myofibroblast differentiation by targeting the core profibrotic pathway, TGF-β1, which could be achieved by blocking the production or activity of the ligands, by preventing receptor activation or by targeting the downstream effectors like the Smad cascade (101–103). It is interesting that, despite the deep understanding of the pathways leading to fibrosis and the mechanisms and pathogenic actions of fibrotic fibroblasts, the mode of action of one the very few approved anti-fibrotic drugs, pirfenidone, remains poorly understood. It is believed that effects on the TGF-β1 pathway and consequently on proliferation, myofibroblast differentiation and collagen deposition are involved (104). The other approved anti-fibrotic drug, nintedanib, is a non-specific tyrosine kinase inhibitor, which prevents the phosphorylation multiple targets involved in the development of fibrosis including platelet-derived growth factor receptor (PDGFR), fibroblast growth factor receptor (FGFR), vascular endothelial growth factor receptor (VEGFR) as well as the type II TGF-β receptor (105, 106). Examples of other pathways that may prevent the activation of pathogenic fibroblasts or reduce their recruitment include the inhibition of the Notch (107), JAK/STAT (108), TNF receptor (109), lysophosphatidic acid receptor 1 (LPA_1_) (110), RANK ligand (42) or Ca^2+^ signalling (111) pathways, and on the other hand, the activation of the Wnt pathway (112, 113).

Targeting fibroblast mechanotransduction pathways is another option. Mechanical forces applied to connective tissues alter the gene expression pattern and activity of fibroblasts, as mechanical signals are converted into intracellular signalling events, a process called mechanotransduction. As mechanical stress produced by matrix stiffness can lead to fibrosis, strategies targeting these pathways are also under development. This can be achieved, for example, by using compounds that inhibit the FAK pathway, αV integrins or the YAP/TAZ transcription factors, among others (114). A different approach to control the mechanical activation of fibroblasts with application in the prevention of fibrotic encapsulation consists of modulating the stiffness of the materials used in the implants (115, 116).

Myofibroblasts present with increased survival due to the expression of anti-apoptotic signals. Promoting fibroblast death has shown beneficial outcomes in models of fibrosis, for example with the administration of TNF-related apoptosis-inducing ligand (TRAIL) related compounds like TLY012, which reduced fibrosis in a model of scleroderma (117). Similarly, mimetics of the pro-apoptotic protein BH3, like the small molecule ABT-263 can also override the enhanced survival capacity of myofibroblasts (118).

Inactivation of hyperactivated fibroblasts can also be achieved through the induction of cellular senescence, an approach that has been tested in models of arthritis (16, 119, 120), where fibroblasts exhibit overactivation of their immune component and degradative properties. These strategies, however, might benefit from combining with senolytics, to induce the clearance of these cells once they have exerted their functions. Although the use of senolytics alone are being investigated as anti-ageing approaches (121), their potential off-target effects and how these therapies may affect physiological wound healing have yet to be elucidated.

Given that fibroblast activation is usually preceded by immune cell activation, targeting the stromal-immune crosstalk may also offer opportunities. In many fibroblast driven diseases, an inflammatory response often precedes the activation of myofibroblasts. Then, preventing the crosstalk, i.e., intercepting the messenger, early in the pathogenic process may be a useful strategy. For example, by pre-coating implants using potent anti-inflammatory drugs like steroids, fibrotic encapsulation could be prevented (122, 123), although the use of corticoids may increase the risk of infections. In addition, this strategy can only be applied in situations when the time of initiation of the fibrotic cascade is known. Interestingly, interfering with the crosstalk between immune cells and the ECM using recombinant pentraxin 2, is also under development for the treatment of pulmonary fibrosis (124). In a recent preprint it was shown that promoting the shift from M2 towards M1 macrophage phenotype by targeting CD206 reduced fibrosis in the bleomycin mouse model of pulmonary fibrosis, to a similar extent as pirfenidone and nintedanib (125).

The approaches discussed above are aimed the prevention or de-activation of fibroblast aggressive phenotypes. However, strategies to enhance the actions of these cells can be beneficial in certain conditions where a loss, rather than an excess of fibroblast activity is present. The use of topical vitamin C is a well-known anti-ageing strategy for the enhancement of collagen deposition by dermal fibroblasts (126). The activation of periodontal fibroblasts using fibroblast growth factor 2, FGF-2, can induce tissue regeneration in periodontitis (127). Fibroblasts themselves can be used as therapeutics, which can be given as injections of cell suspensions to induce tendon injury repair (128). A study using fibroblast cell-based therapy also showed prevention of hair loss in a mouse model of alopecia areata (129), in line with other reports suggesting the use of fibroblast growth factors or the TGF-β receptor ligand Scube3 as hair growth stimulants (99, 130). Although still in its infancy, the novel insights provided by single-cell technologies reveal another way to enhance fibroblasts activity, consisting of activating those subpopulations presenting with pro-repair actions, in contrast or in addition to targeting only the aggressive pathogenic populations (47). Theoretically, taking advantage of their plasticity, by promoting the actions of the 'good' fibroblasts, or by inducing the reprogramming of the 'bad' ones, for example by targeting the transcription factor PU.1 (131), may bring novel therapeutic approaches.

Finally, strategies based on promoting our body's endogenous mechanisms to deal with inflammation and restoration of homeostasis have also been developed and tested in fibroblasts and the diseases that they drive. These 'pro-resolving' mechanisms and the evidence for their therapeutic potential for fibroblast mediated diseases will be discussed in the next sections.

## 3 Resolution pharmacology to target fibroblasts

### 3.1 The resolution of inflammation at a glance

Resolution Pharmacology is a type of therapeutic strategy based on the activation of endogenous mechanisms aimed at promoting homeostasis after a pro-inflammatory stimulus (12). These mechanisms consist of several families of receptors and their ligands, expressed in immune cells but also in others like endothelial cells and fibroblasts. Pro-resolving systems include, among others, the formyl peptide receptor 2 (FPR2) and the ligand annexin A1, the melanocortin system, and lipid mediators like lipoxin A4, resolvins or protectins, acting on a wide range of receptors (11, 13, 132, 133).

All these systems were discovered and extensively studied upon the realization that inflammatory responses are brought down to homeostatic levels by the active engagement of endogenous pathways rather than passively as previously believed (134). The physiological inflammatory response comprises a balanced onset of inflammation, efficient elimination of pathogenic agents and smooth transition into the termination phase culminating in the restoration of homeostasis, during which resolution mechanisms play a major role. A second important turning point in the field, with novel therapeutic implications, is the appreciation that chronicity may emerge from persistent and exacerbated pro-inflammatory signals as well as from the failure of the resolution pathways, a concept now well accepted for example to explain the chronicity of rheumatoid arthritis where aggressive fibroblasts are key players (9, 10, 41). This represented the beginning of a new field of research, which in less than 15 years since its formal birth in 2007 (11), has delivered several drugs approved for use in humans, and many others under clinical development (12, 135).

Several mechanisms of resolution have been described, like the active reduction in cytokine release and prevention of cell recruitment, enhancement of macrophage efferocytosis, induction of macrophage class switch, promotion of granulocyte apoptosis, and the production of anti-inflammatory cytokines (136–138). Pro-resolving mediators are also involved, not only in the active termination of the inflammatory response, but in the activation of the repair mechanisms and wound healing (139–141). Given their involvement in driving pathogenic actions, together with their active role during healing and repair, the therapeutic potential of pro-resolving mediators has been tested in fibroblasts and models of fibrosis.

### 3.2 Resolution pharmacology to target fibroblasts mediated diseases

everal mediators and their derivatives, acting on various pro-resolving receptors have been tested in fibroblasts and in animal models of fibrosis. For example, the FPR2 synthetic peptide agonist WKYMVm reduced the accumulation of myofibroblasts and macrophage infiltration in the bleomycin model of skin fibrosis (142). The endogenous ligand for FPR2, annexin A1, has also shown beneficial effects in reducing fibrosis in a mouse model of non-alcoholic steatohepatitis (NASH) (143), while the Annexin A1 derived peptide Ac2-26 reduced collagen deposition in a model of silicosis (144). Lipoxin A4, a bioactive lipid derived from arachidonic acid, the receptor for which is also FPR2, was shown to reduce cell proliferation and activation in lung fibroblasts from human (145) and from mouse (146). This lipid mediator also reduced IL-1β, IL-6 and metalloproteinase 3 expression in synovial fibroblasts, while increasing the metalloproteinase inhibitor TIMP1 (147). Activation of FPR2 with the synthetic small molecule 'compound 43' showed reduction in fibroblast proliferation as well as increased apoptosis using *in vitro* cultured human synovial fibroblasts (148). Importantly, expression of the receptor FPR2 has been confirmed in synovial fibroblasts from rheumatoid arthritis patients (149, 150). Another peptide, C15, derived from the endogenous pro-resolving protein chemerin, which binds to the receptor ChemR23, was tested in a mouse model of skin wounds, showing improved collagen organisation and fibre alignment (151).

The anti-fibrotic actions of several specialized pro-resolving mediators (SPMs), which are bioactive molecules produced from essential polyunsaturated fatty acids, have also been tested using *in vitro* and *in vivo* models. For example, resolvins D1 and E1 were able to reduce inflammatory responses in cardiac fibroblasts (152). In line with this, the endogenous expression of the enzyme arachidonate 5-lipoxygenase (ALOX5), involved in the synthesis of SPMs, was found to be essential in containing the damage occurring upon myocardial infarct. ALOX5 and ALOX15, other lipoxygenase enzymes involved in the biosynthesis of SPMs, were also identified in rheumatoid arthritis synovium (153). Achilles tendinopathy derived fibroblasts showed a reduced pro-inflammatory aggressive phenotype after treatment with 15-epi-Lipoxin A4 or maresin 1 (154). Maresin 1 was also able to reduce proliferation in lung fibroblasts (155). SPMs also showed beneficial effects using an *in vivo* pulpitis model, as well as dental pulp fibroblasts, where inhibition of NFκB and subsequent reduction in inflammatory responses were observed (156). Protectin DX is another SPM derived from docosahexaenoic acid. Using a model of acute lung injury, protectin DX inhibited TGF-β1 induced proliferation of fibroblasts, myofibroblast differentiation and collagen deposition (157).

Collectively, these reports provide evidence for the potential application of pro-resolving mediators as well as synthetic derivatives for conditions where fibroblasts play a pathogenic role. In addition to the previous examples, extensive research has been conducted using pro-resolving mediators targeting the melanocortin pathway. These will be discussed in detail in the next section.

## 4 Targeting the fibroblast with melanocortin drugs

### 4.1 The melanocortin system at a glance

The peptides adrenocorticotropic hormone (ACTH), α-, β-, and γ-melanocyte-stimulating hormones (α, β, γMSH) are the four endogenous melanocortin peptides derived from the enzymatic processing of a larger precursor termed proopiomelanocortin protein (POMC). Two other melanocortin ligands have been identified, in this case encoded by individual genes, agouti related neuropeptide (AGRP) and agouti signalling protein (ASIP). While the POMC-derived peptides exert agonistic actions, ASIP and AGRP act mostly by antagonising the actions of the former (13). The five receptors that these ligands use to exert their functions (MC_1-5_) belong to the class A rhodopsin-like G-protein coupled receptors (GPCR), a fact that makes melanocortin receptors appealing druggable targets, as roughly 35% of all currently approved drugs act by targeting a GPCR (158).

**Figure 2:**
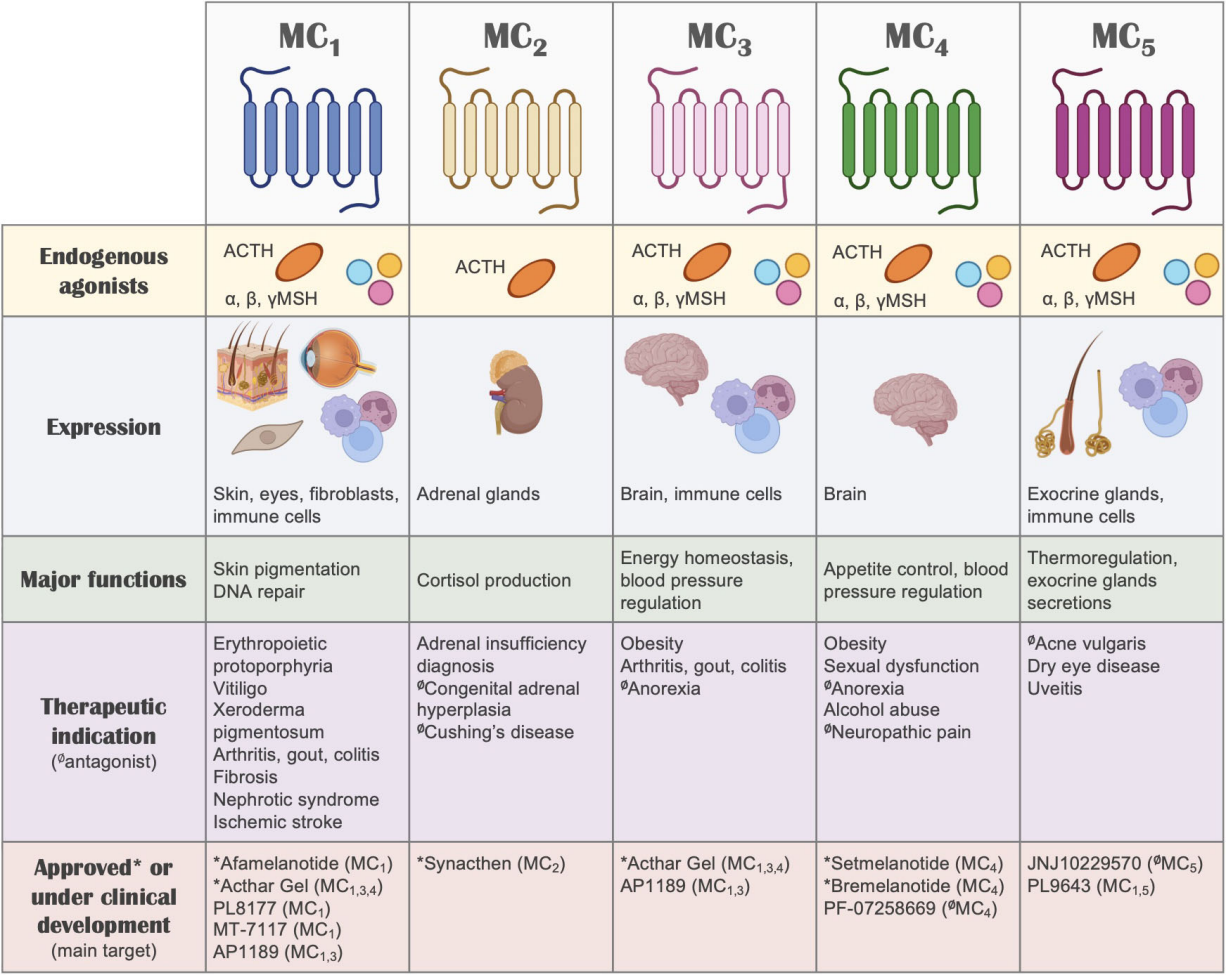
The melanocortin system. A summary of the melanocortin system is shown, including receptors, ligands, tissue distribution, their biological actions, potential therapeutic indication and drugs that are currently under clinical development or approved for use in humans. Regarding therapeutic indications, the receptor that mediates the therapeutic actions is highlighted, without implying receptor selectivity. Unless indicated otherwise (with the symbol Ø denoting antagonism), the molecules included in this summary are melanocortin receptor agonists.

Besides their GPCR nature and the associated implications for drug discovery, one of the most interesting aspects of the melanocortin system (**Figure 2**) that makes it very attractive for the development of new therapies is the remarkable functional specialization of the MC receptors, controlling multiple and diverse processes around the body, including skin pigmentation and DNA repair, appetite regulation, blood pressure, production of cortisol, regulation of glands secretions, and immune responses, among others (13). This specialization also derives from the specific tissue distribution of each receptor, the differential processing of the POMC precursor to yield the different agonists, and the expression in certain tissues of the antagonists, which for instance, can also act as biased agonists (159). All these aspects allow for a fine-tuned regulation of the actions that the melanocortin system controls, while simultaneously offering multiple ways to target and intervene the system for therapeutic purposes. We recently reviewed elsewhere the vast array of therapeutic opportunities that the targeting of each one of these receptors offers, not only as a hope for the future, but as a current success, as several MC drugs have already been approved and many others are in clinical phase of development (135).

Here we focus on exploring and revising the existing evidence for the use of melanocortin based therapies for the management of fibroblasts mediated conditions.

### 4.2 Fibroblasts are target and source of melanocortin components

Various studies have reported the expression of components of the MC pathway in fibroblasts, including receptors and ligands. On *in vitro* cultured cells, expression of MC_1_ has been detected in human dermal fibroblasts at the gene expression level (160, 161) as well as at the protein level using immunofluorescence (160). Similarly, synovial fibroblasts obtained from osteoarthritis patients undergoing knee replacement also show immunoreactivity for MC_1_ (162). The expression of other MC receptors (*MC3R*, *MC4R*, *MC5R*) was also detected by real-time PCR in synovial fibroblasts obtained from rheumatoid arthritis patients, although at lower levels compared to *MC1R* (16). Other fibroblast populations expressing MC_1_ include dermal papillae and connective tissue sheath fibroblasts (163).

The expression of MC_1_ has also been detected in tissue samples, although the cellular localisation specifically on fibroblasts has not always been fully inferred, as MC_1_ is highly expressed in melanocytes and other cell types found in complex tissues. For example, immunohistochemical detection of MC_1_ was shown in normal skin samples from healthy volunteers, locating in dermal and epidermal regions. Interestingly, the expression of MC_1_ was highly increased in skin samples from acute burn injury, but essentially absent in skin samples obtained from keloid scars (164). Congruently, fibroblasts derived from keloid scars were unresponsive to the anti-fibrotic effects of αMSH, in line with the absence of the target. Intriguingly, this fibrotic condition, characterised by enlarged raised scars that spread beyond the limits of the original wound area, is more common in individuals with dark skin (165), which are more likely to carry a fully functional wild-type form of the *MC1R* gene, compared to individuals with lighter skin. However, the link between this receptor and the formation of keloids has yet to be fully elucidated. A different study confirmed the increased expression of MC_1_ in acute burns (166). Although the authors did not detect the expression of MC_1_ in healthy skin, MC_1_ and the ligand αMSH were increased in samples from hypertrophic scars. In addition, using a mouse model of cutaneous wounds, they observed increased expression of both MC_1_ and αMSH, with a peak of expression at day 3 post-injury, and declining by day 21.

MC_1_ protein has also been detected in tissues of scleroderma patients obtained from clinically involved skin areas. MC_1_ was detected in various dermal cells including endothelial cells, macrophages and fibroblasts, the latter suggested by the co-expression with prolyl 4-hydroxylase subunit beta (P4Hβ). In this case, there were no differences in MC_1_ expression levels in disease compared to healthy skin (167).

In addition to receptors, the endogenous production of melanocortin ligands by fibroblasts has been reported. Besides αMSH increasing during cutaneous injury as mentioned earlier, the precursor POMC protein can be detected at gene expression level in dermal fibroblasts (160), while cultured synovial fibroblasts are able to produce and release the melanocortin agonist ACTH (16). ACTH and αMSH are also detected in supernatants from human dermal fibroblasts (168), with enhanced expression when cells are stimulated with TNF-α (169). In contrast, reduced levels of POMC are detected when fibroblasts are stimulated with TGF-β1 (170). Accordingly, expression of the POMC processing enzymes PC1 and PC2 can be detected in human dermal fibroblasts (171). In the skin, the release of melanocortin peptides can be triggered by exposure to UV radiation. However, the existence of a local 'hypothalamic-pituitary-adrenal axis' in the skin has been proposed upon the discovery of the ability of the skin to produce corticotropin-releasing hormone under stress conditions (172). The expression of the receptor MC_2_, which is normally found almost exclusively on the adrenal glands, was found dysregulated in scalp skin samples from alopecia areata, suggesting a deficit for ACTH/MC_2_ axis in the pathogenesis of this condition (173).

Altogether, these reports demonstrate, not only the presence of MCRs in multiple types of fibroblasts, but their active involvement in the endogenous control of their functions, suggesting MCRs, and particularly MC_1_, as plausible therapeutic targets for the treatment of fibroblast-mediated conditions.

### 4.3 Melanocortin actions on fibroblasts

**Figure 3:**
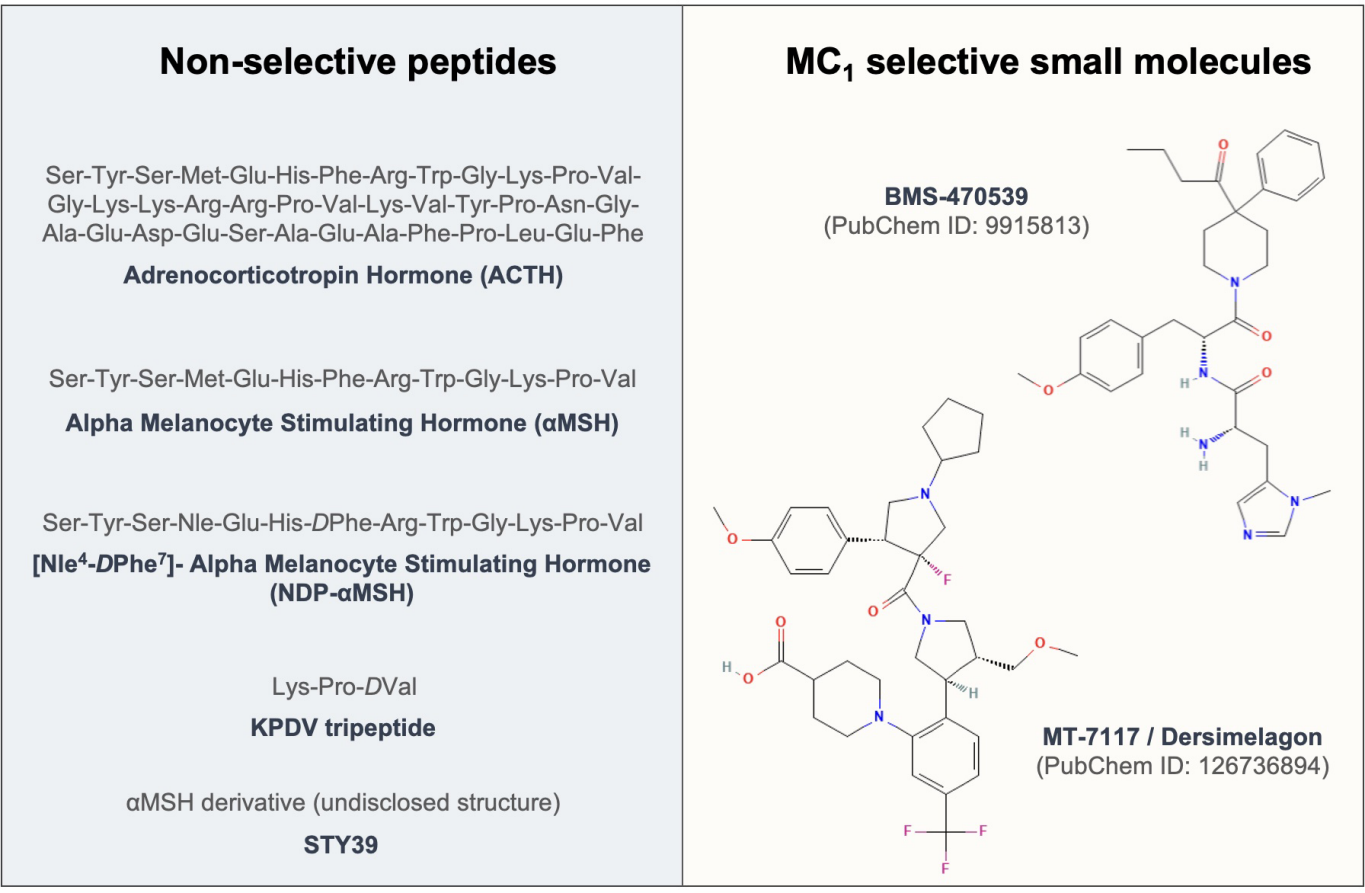
Sequences and structures of melanocortin drugs tested in fibroblasts and related diseases. The amino acid sequences and chemical structures of the melanocortin agonists that have been tested using *in vitro* and *in vivo* models of diseases mediated by fibroblasts included in **Tables 1**, **2** are shown.

The first evidence of testing the effects of melanocortins on fibroblasts dates back to 1961 (174), where ACTH was used to address its effects on fibroblast growth. To date, enough evidence has been generated to support the protective role of these compounds (see **Figure 3**) in models of fibrosis and other fibroblast-mediated conditions, resulting so far in the approval of a clinical trial testing the efficacy of an MC_1_ selective molecule MT-7117 (also known as dersimelagon) for the treatment of diffuse cutaneous systemic sclerosis.

Studies conducted using *in vitro* models of fibroblast activation are summarised in **Table 1**. Most of the work has been conducted with human primary dermal fibroblasts in which the effects of the endogenous peptide αMSH were tested. Collectively, these studies show that αMSH was able to reduce collagen synthesis (160, 161) while activating MMP-1 (183), suggesting the acquisition of a remodelling phenotype, relevant for treating fibrotic conditions. αMSH also reduced activation of fibroblasts, as measured by αSMA staining (161), reduced inflammatory markers like TNF-α-induced activation of NFκB (179) or IL-1β-induced IL-8 release (181), as well as it conferred cytoprotection under inflammatory stimuli like IL-1β, LPS and TNF-α (180). The effects of αMSH on fibroblasts obtained from keloid scars were also addressed, resulting in lack of efficacy derived from the reduced or negligible expression of the target MC_1_ on these cells. More recently, it was suggested that strategies restoring MC_1_ expression in keloid fibroblasts by treating these cells with a long noncoding RNA molecule, LINC00937, resulted in suppressed ECM deposition and cell proliferation (184).

**Table 1:** Testing of melanocortin drugs using *in vitro* models of fibroblast activation.

*In vitro* model	MC compound	Biological effect	Translational relevance	Year (Reference)
Scleroderma human dermal fibroblasts	MT-7117	↓ αSMA^+^ cells ↓ Collagen	Scleroderma	2022 (167)
Thyroid eye disease orbital fibroblasts	αMSH	↓ IL-6, IL-8 MCP-1, ICAM-1, COX2	Thyroid eye disease (Grave's disease)	2021 (175)
Rheumatoid arthritis human synovial fibroblasts	BMS-470539	↓ Proliferation ↓ Aggressive profile ↓ Collagen	Rheumatoid arthritis	2020 (16)
Human lung fibroblasts	αMSH	↓ Proliferation ↓ αSMA^+^ cells	Lung fibrosis	2018 (176)
Osteoarthritis human synovial fibroblasts	αMSH BMS-470539	↓ Cytokines IL-6, IL-8 ↑ Cell adhesion	Osteoarthritis	2016 (162)
Human dermal fibroblasts, normal and keloid scars	αMSH	↓ Collagen ↓ Metabolic activity ↓ αSMA^+^ cells	Dermal fibrosis, keloid scars	2013 (164)
Human Tenon’s capsule fibroblasts	αMSH	↓ Proliferation ↓ Collagen ↓ Cytokines	Conjunctival fibrosis	2012 (177)
Human dental pulp fibroblasts	PGA-αMSH	↑ Proliferation ↑ Cell adhesion ↓ IL-6, TNF-α	Endodontic regeneration	2010 (178)
Human dermal fibroblasts	αMSH NDP-αMSH ACTH	↓ Collagen	Dermal fibrosis	2009 (161)
Human dermal fibroblasts	αMSH KPDV	↓ NFκB ↓ ICAM-1	Skin inflammatory conditions	2006 (179)
Human dermal fibroblasts	αMSH	↑ Cytoprotection ↓ Apoptosis	Skin inflammatory conditions	2005 (180)
Human dermal fibroblasts	αMSH	↓ Collagen	Dermal fibrosis	2004 (160)
Human dermal fibroblasts	αMSH	↓ IL-8 (IL-1β stimulated) ↑ IL-8 (unstimulated)	Skin inflammatory conditions	1999 (181)
Human dermal fibroblasts	αMSH	↑ IL-8	Skin inflammatory conditions	1999 (182)
Human dermal fibroblasts	αMSH	↑ MMP-1	Photo-ageing	1995 (183)

Reports on the effects of melanocortin drugs using *in vitro* models of fibroblast activation are shown in chronological order, showing the most recent first. The arrow pointing up (↑) means "increase", the arrow pointing down (↓) means "decrease".

In primary lung fibroblasts, αMSH reduced αSMA expression and cell proliferation (176), while on Tenon's capsule fibroblasts, involved in conjunctival fibrosis, αMSH reduced collagen deposition, proliferation and cytokine release (177). A modified αMSH, covalently coupled to poly-L-glutamic acid (PGA-αMSH), showed beneficial effects for endodontic regeneration due to its ability to induce cell adhesion, proliferation and reduction of IL-6 and TNF-α on dental pulp fibroblasts. Using orbital fibroblasts from patients with Grave's disease, it was shown that αMSH can also reduce the inflammatory phenotype of these cells (175).

The effects of melanocortin treatment, in this case using the selective MC_1_ agonist BMS-470539, on synovial fibroblast have been addressed using cells from both osteoarthritis (OA) and rheumatoid arthritis (RA) patients. In OA-derived fibroblasts, the compound increased cell adhesion and reduced the release of pro-inflammatory cytokines (162). Similar protective effects were detected in RA fibroblasts, where the compound reduced proliferation and attenuated the aggressive phenotype typically exhibited by these cells although in this case, the acquisition of a senescence-like phenotype was also observed (16). This unique phenotype resembled that of a remodelling phase of wound healing, with decreased collagen and an increase in remodelling enzymes, indicating that this effect may also have applications in fibrotic diseases.

An interesting aspect related to the therapeutic use of melanocortins is the impact of genetics on the actions of these drugs. It was found that the ability of αMSH in reducing dermal fibroblast proliferation was diminished by the presence of *MC1R* gene variants like R163Q, R151C and V60L (185), strongly suggesting the need to incorporate pharmacogenomics approaches applied to MC_1_-based therapies to deliver personalised therapies.

The mechanisms and pathways behind the actions exerted by melanocortins on fibroblasts are not well studied. While the canonical signalling pathway engaged upon MCR activation is the increase in cyclic-AMP, it was found that inhibition of human lung fibroblast proliferation and differentiation by Gs-coupled receptors is not predicted by the magnitude of cAMP response (176), suggesting that the involvement of other pathways should be investigated to understand these actions. Indeed, ERK1/2 phosphorylation was suggested as the pathway mediating the actions of BMS-470539 on RA synovial fibroblasts (16). In terms of downstream signalling pathways, it has been suggested that the modulation of the inflammation-related transcription factors NFκB and AP-1 may be involved in the antifibrotic actions of melanocortin peptides (181).

Much of the work described above has been generated by Böhm and colleagues, who also provided useful insights into the actions of these drugs using *in vivo* models of fibrosis (**Table 2**). In a mouse model where skin fibrosis was induced by intracutaneous injections of TGF-β1, αMSH reduced the accumulation of αSMA positive cells and collagen deposition (160). In one the most commonly used models of skin fibrosis induced by intracutaneous injections of bleomycin, αMSH also showed potential therapeutic effect by reducing skin thickness, collagen content and expression of superoxide dismutase 2 (SOD2) and heme oxygenase 1 (HO-1) (161). Later, using a low dose of bleomycin (10ug/mouse daily), the authors found that while wild-type C57BL/6J mice did not show signs of fibrosis at that dose, *Mc1r* ^e/e^ mice, deficient for MC_1_ receptor on a C57BL/6J background, displayed obvious signs of fibrosis like increased skin thickness, collagen expression and levels of chemoattractant protein MCP-1, suggesting that the deficiency in *Mc1r* increases the susceptibility to develop bleomycin-induced fibrosis (194). This is of relevance due to the high polymorphic nature of *MC1R* gene, for which loss-of-function variants are common in certain populations like north Europeans. Indeed, there is epidemiological evidence that highlights a possible association between variants in the *MC1R* gene and the risk of developing fibrosis. MC_1_ is involved in the regulation of pigmentation, and several *MC1R* variants are known to be associated with fairer skin, hair and eye colour (196, 197). In a cohort of idiopathic pulmonary fibrosis patients, a higher disease incidence was noted in white patients compared to black patients (198). Similarly, in a recent observational study, it was highlighted that there were three times as many idiopathic pulmonary fibrosis patients with lighter eye colour compared to those with dark eye colour (199).

**Table 2:** Testing of melanocortin drugs using *in vivo* models driven by fibroblasts.

In vivo model	MC compound	Biological effect	Translational relevance	Year (Reference)
Bleomycin induced skin fibrosis (mouse)	MT-7117	↓ αSMA^+^ cells ↓ Skin thickness ↓ Inflammation	Scleroderma	2022 (167)
K/BxN serum transfer induced arthritis (mouse)	BMS-470539	↓ Leukocyte infiltration ↓ Paw swelling ↓ Clinical score	Rheumatoid arthritis	2020 (16)
Skin injury (mouse)	αMSH	↓ Fibroblast number ↓ Scar ↑ Collagen organization	Cutaneous wound healing	2015 (186)
Partial hepatectomy (rats)	NDP-αMSH	↑ IL-6/SOCS pathway	Liver regeneration	2013 (187)
Bleomycin induced lung injury (mouse)	STY39	↓ Collagen, hydroxyproline ↓ αSMA^+^ cells ↓ Leukocyte infiltration ↓ IL-6, TNF-α, MIP-2	Pulmonary fibrosis	2011 (188)
Bleomycin induced skin fibrosis (mouse)	αMSH	↓ Collagen ↓ Oxidative stress	Dermal fibrosis	2009 (161)
Bleomycin induced lung injury (rat)	NDP-αMSH	↓ TGF-β1, iNOS ↓ IL-6, TNF-α, ↓ CCL-2, CCL-5	Acute lung injury	2007 (189)
Carbon tetrachloride (CCl4) induced hepatic fibrosis (mouse)	αMSH	↓ Collagen ↓ TGF-β1, COX2 ↓ αSMA^+^ cells ↑ MMP activity	Liver fibrosis	2006 (190)
Thioacetamide induced liver fibrosis (mouse)	αMSH	↓ Matrix density ↓ TGF-β1, COX2 ↑ MMP activity ↓ TIMP activity	Liver fibrosis	2006 (191)
TGF-β1 induced skin fibrosis (mouse)	αMSH	↓ αSMA^+^ and vimentin^+^ cells ↓ Collagen	Dermal fibrosis	2004 (160)
Cyclosporine induced tubulointerstitial fibrosis (rat)	αMSH	↓ Collagen ↓ Apoptosis ↓ Inflammation	Tubulointerstitial fibrosis	2004 (192)
Partial hepatectomy (rat)	αMSH	↑ Liver protein content	Liver regeneration	1975 (193)
**Insights from gene deficiency**				
Bleomycin induced skin fibrosis (mouse, *Mc1r* ^e/e^)	MC_1_ deficiency	↑ Collagen, skin thickness ↑ MCP-1, CTGF	Points at MC_1_ as therapeutic target	2014 (194)
High fat diet (mouse, *Mc4r* ^-/-^)	MC_4_ deficiency	Steatohepatitis ↑ Leukocyte infiltration ↑ Fibrosis	Points at MC_4_ as therapeutic target	2011 (195)

Reports on the effects of melanocortin drugs using *in vivo* models of fibroblast activation are shown in chronological order, showing the most recent first. The arrow pointing up (↑) means "increase", the arrow pointing down (↓) means "decrease".

These encouraging results regarding the potential anti-fibrotic actions of melanocortins, and likely the potential for MC_1_ receptor targeting receptor targeting for skin fibrosis, possibly prompted the initiation of the first clinical trial testing a melanocortin compound for the treatment of a fibrotic disease like systemic sclerosis. The efficacy and tolerability of MT-7117 (reported as MC_1_ selective) are being tested in a phase II, randomized, double-blind, placebo-controlled trail, using an orally administered formulation for 52 weeks. Previous positive antifibrotic actions of this compound were observed in the bleomycin-induced skin fibrosis model in mice (167). The trial is currently on recruitment phase and efficacy parameters that will be measured include skin thickness (modified Rodnan Skin Score, mRSS), ACR CRISS score and disability questionnaires like HAQ-DI, among other outcomes (ClinicalTrials.gov identifier NCT04440592).

Bleomycin is also used to induce fibrosis in the lungs by administering a single intratracheal instillation of bleomycin solution. Two αMSH derivatives, STY39 and NDP-αMSH were tested in mice and rats, respectively, using this model of lung injury (188, 189). Both compounds demonstrated their ability to reduce the expression of pro-inflammatory mediators like IL-6, TNF-α and TGF-β1. In addition, STY39 displayed anti-fibrotic actions like reduced collagen and hydroxyproline content, reduced myofibroblast number and tissue damage, and increased survival.

Several studies have been conducted in the context of liver fibrosis, suggesting that αMSH reduces markers of fibrosis in the carbon tetrachloride (CCl4) and in the thioacetamide-induced mouse models of liver fibrosis (190, 191). Interestingly, mice deficient for the melanocortin receptor type 4 fed on a high fat diet develop signs of steatohepatitis associated with obesity, insulin resistance, dyslipidaemia, inflammatory cell infiltration, liver fibrosis, and the development of hepatocarcinoma after one year. Thus, *Mc4r* ^-/-^ mice on a high-fat diet were proposed as a model of non-alcoholic steatohepatitis, or NASH (195). The effects of αMSH and its derivative NDP-αMSH in tissue regeneration upon partial hepatectomy were also investigated, showing some positive outcomes as measured by modulation of the IL-6 pathway and protein content (187, 193).

Cutaneous wound healing, using a mouse model in which through-and-through wounds were inflicted in the dorsal area, was also improved with αMSH administered 30min before creating the wounds. The drug reduced immune cell infiltration, fibroblast number, scar formation, and improved collagen fibres organization (186). Similarly, αMSH presented positive results in a model of renal fibrosis, induced by the administration of cyclosporine A, including reduction in TGF-β1, inflammatory markers, cell apoptosis and collagen deposition (192). Finally, the effects of the selective MC_1_ compound BMS-470539 showed signs of reducing the aggressive activity of synovial fibroblasts, an effect that translated into anti-arthritic actions *in vivo*, using the model of K/BxN serum transfer induced arthritis in mice (16).

## 5 Challenges are directions towards melanocortin-based therapies targeting fibroblasts

More than 60 years after the first report testing the role of the endogenous melanocortin ACTH on fibroblasts, a synthetic agonist is being trialled in a phase II study for the treatment of systemic sclerosis. In between, substantial evidence has been generated to support the therapeutic potential of melanocortin agonists for the treatment of fibroblast driven diseases, including fibrosis and more.

Besides the recent success of the melanocortin field in reaching clinical development and drug approvals (135), several challenges and hurdles still exist, some difficult to solve, others at our immediate reach. One of the major handicaps in MC research has been the lack of validated antibodies with demonstrated selective detection of each specific receptor subtype. This, together with the scarcity of true selective agonists, has prevented the progress towards obtaining an accurate understanding of the cellular and tissue distribution of these receptors as well as their specialised functions. The single-exon nature of the genes encoding for the MCRs also presents a challenge in determining gene expression accurately as samples require harsh DNAse treatments, not always conducted in all studies. Then, investing in producing better tools will help to achieve substantial advances within the field. A possible avenue could be formation of a university/commercial partner consortium to fund generation of these tools, which then will be available to the wider community.

The early discovery in 2002 of the MC_1_ selective compound BMS-470539 (200) together with the use of mice deficient in *Mc1r* may have helped to point to a role for MC_1_ in fibroblast functions, as discussed earlier in section 4.3. However, the role and the therapeutic potential of other MCRs to target fibroblasts should not be excluded.

An effort from the MC community is also needed to improve accuracy and prevent confusion, and the accompanying distrust, when reporting drug activity. For example, setmelanotide is commonly wrongly referred to as an MC_4_ agonist, when this compound is not selective for this receptor, or melanotan I and II are usually alluded to as MC_1_ selective peptides when they are pan-agonists. Equally needed is the characterization of novel MC compounds according to several signalling pathways, instead of the classical approach based solely on Gs protein derived cAMP accumulation. It is now accepted that biased signalling can derive from engagement with MC receptors, as for other GPCRs, through the activation of alternative pathways other than the canonical cAMP. This should be considered, not only for the sake of accuracy and to avoid mischaracterization of drugs, but because the existence of ligand bias provides interesting therapeutic opportunities to achieve functional selectivity. Indeed, the novel melanocortin candidate AP1189, currently in phase II trials, may have been discarded early during development as it does not induce cAMP, but activates ERK1/2 phosphorylation instead (15). This translates into anti-inflammatory activity, derived from the latter, with no unwanted melanogenic activity, derived from the former. Moreover, as previously mentioned, cAMP magnitude does not correlate with fibroblast responses (176), indicating that at least when addressing the anti-fibroblast activities of MC compounds, other pathways should be considered.

The possibility of differences between mouse and human receptors needs to be considered during the development of novel MC candidates. For example, a compound demonstrating selectivity at the human MC_3_ receptor may not replicate this activity in the mouse ortholog, as it occurs with the peptide γ_2_MSH (201). Another example of discrepancy between mouse and human receptors is the high constitutive activity presented by the mouse but not for the human MC_1_. This is demonstrated by the phenotype associated with POMC deficiency (i.e. lack of MC ligands): while individuals carrying a mutation in this gene present with fair skin and red hair (202), *Pomc* ^-^/^-^ mice on a black coat mouse strain (129;B6) are still black, due to the high ligand independent activity of this receptor (203).

Other challenges related to fibroblast biology also need to be addressed to achieve optimal translation of melanocortins, as well as other drugs, for the treatment of diseases driven by these cells. For example, a better understanding of the pathogenic mechanisms that are specific to each condition, which contribute to the sustained unresolved activation, as well as how fibroblasts interact with surrounding cells, both immune and others. While in broad terms we now know that excessive collagen deposition drives fibrosis and excessive immune component mediates arthritis, the exact pathogenic mechanisms of fibroblasts in other contexts like fibromyalgia or frozen shoulder are not so well understood and are fundamental to know how to target these cells therapeutically and how to shift them back to homeostatic states.

Another emerging idea is that a general 'anti-fibroblast strategy' may not suffice, and a better definition of fibroblast heterogeneity together with the specialised functions of the different subsets is needed to fine-tune potential fibroblast-based therapeutic approaches. Then, the identification of specific fibroblast subpopulations across organs and tissues, in health and disease, and importantly, their specific functions will provide insights for the development of novel therapies. Collective efforts and initiatives like the Human Cell Atlas (https://www.humancellatlas.org), powered by single-cell technologies, will provide access to a catalogue of all cell types and their subtypes for the whole of the human body. Recently, a stromal atlas constructed from four chronic inflammatory diseases revealed the existence of two major fibroblasts subpopulations, the CXCL10^+^CCL19^+^ inflammatory fibroblasts, and the SPARC^+^COL3A1^+^ perivascular fibroblasts (6).

Irrespective of which specific receptor mediates the actions and what intracellular signalling mechanism may be involved, it has been demonstrated that MC drugs are generally safe for their use in humans (135). In terms of their potential in treating fibroblast mediated disorders, it would be interesting to accurately describe which receptors are expressed, what are their endogenous functions, and whether MC drugs may differentially affect certain fibroblast subpopulations. Their presence in most organs and tissues, their active role in driving disease, their high plasticity, and a better understanding of their functions, make this cell type a very attractive therapeutic target. To date, most of the *in vitro* and *in vivo* evidence for the potential of MC drugs in fibroblast-driven diseases have been conducted using the natural agonist αMSH, but the time is ripe to move forward into testing improved synthetic analogues and small molecules with higher pharmacokinetic and pharmacodynamic profiles to provide hopes for the effective management of countless diseases with high needs involving dysregulated fibroblasts.

## Author contributions

TM-M conceived article structure, searched for information and wrote manuscript. NK, MS and CD-W searched for information and drafted sections. NK and MS contributed equally to this work. All authors contributed to the article and approved the submitted version.
